# Supplementary material for: Differential Transcriptional Programs Reveal Modular Network Rearrangements Associated with Late-Onset Alzheimer’s Disease
Source: Int J Mol Sci. 2025 Mar 6;26(5):2361. doi: 10.3390/ijms26052361 (PMC11900169; doi:10.3390/ijms26052361)
Supplement: Supplementary file 1 [file ijms-26-02361-s001.zip › ijms-3481988-supplementary.pdf]

Supplementary materials

Differential Transcriptional Programs Reveal Modular Network Rearrangements Associated with Late-Onset Alzheimer's Disease

1 RNA Quality control

The quality control of the expression matrices was performed with the NOISeq package [25]. Following QC, the number of features was reduced to 28,263, as low-quality or unreliable features were filtered out. The expression levels across samples became more consistent and the expression biases dependent on GC and length were reduced, as evidenced by the more uniform expression patterns in the bins (See FigureS1). Together, this is a sign that technical artifacts may have been corrected.

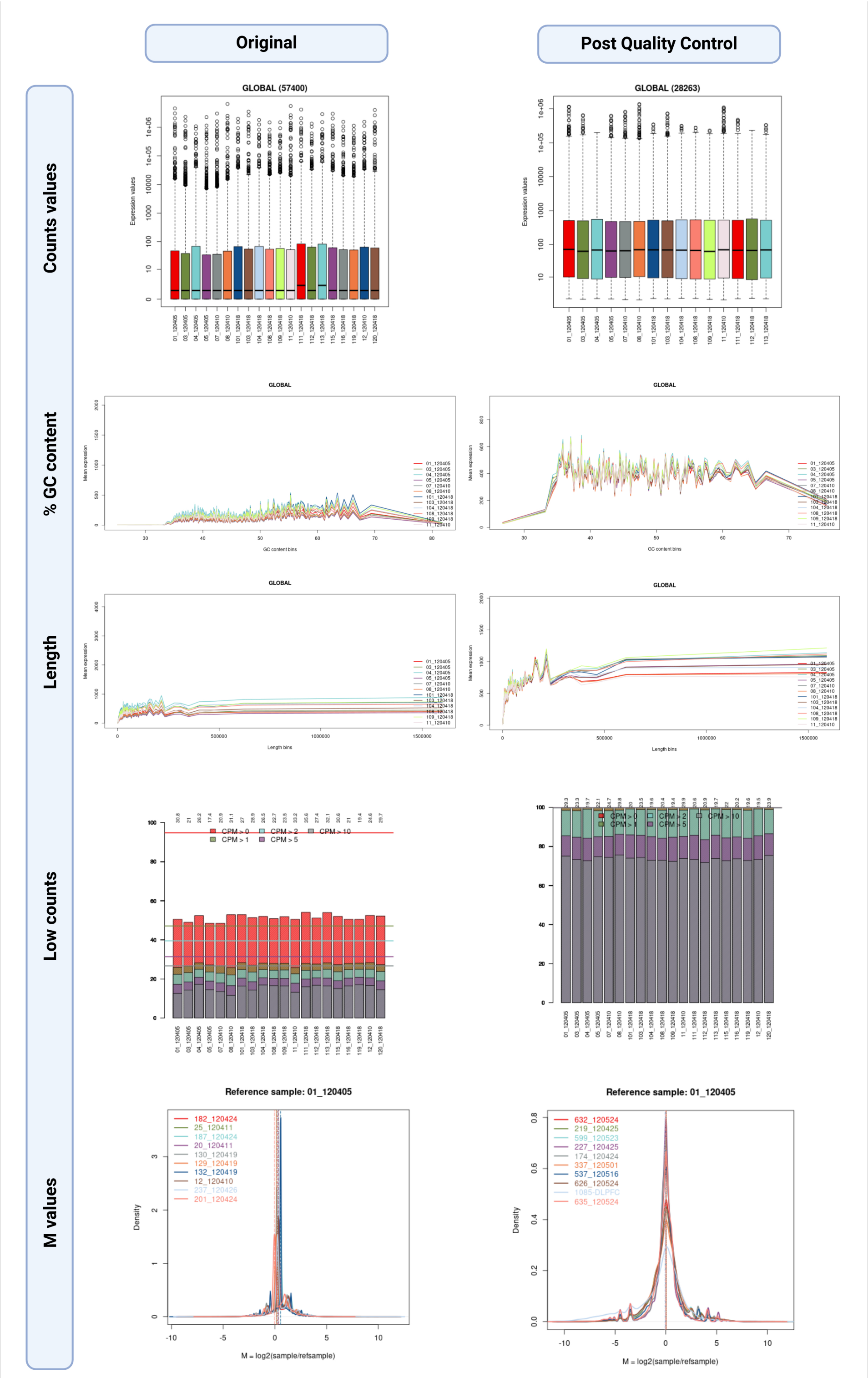

Figure S 1: Plots pre and post-quality control of the RNA-seq counts with NOISeq package. The post-QC plots suggest that technical artifacts have been successfully mitigated, resulting in more reliable and consistent data. Created in Biorender.com

2 Metrics by percentile

To understand the behavior of the network across different thresholds based on correlation intensity, we analyze its structural changes at various percentiles. As the network is pruned by percentile (See Supplementary Figure S1), its structure fragments, increasing the number of disconnected components. This fragmentation peaks at intermediate percentiles, where enough connections are removed to form isolated subgroups. However, at extreme percentiles, the number of components decreases again. In lower percentiles, the network is more dispersed with weakly connected nodes, leading to a low clustering coefficient. As less connected nodes are filtered out, the remaining structure becomes denser, peaking in clustering at the 99.99th percentile. Beyond this, excessive pruning removes key connections, reducing clustering and fragmenting the network. The largest component retains most genes across percentiles but collapses at the highest levels of filtering. The 99.99th percentile is particularly useful for identifying the network's backbone, pointing out hubs and robust connections that define its fundamental structure.

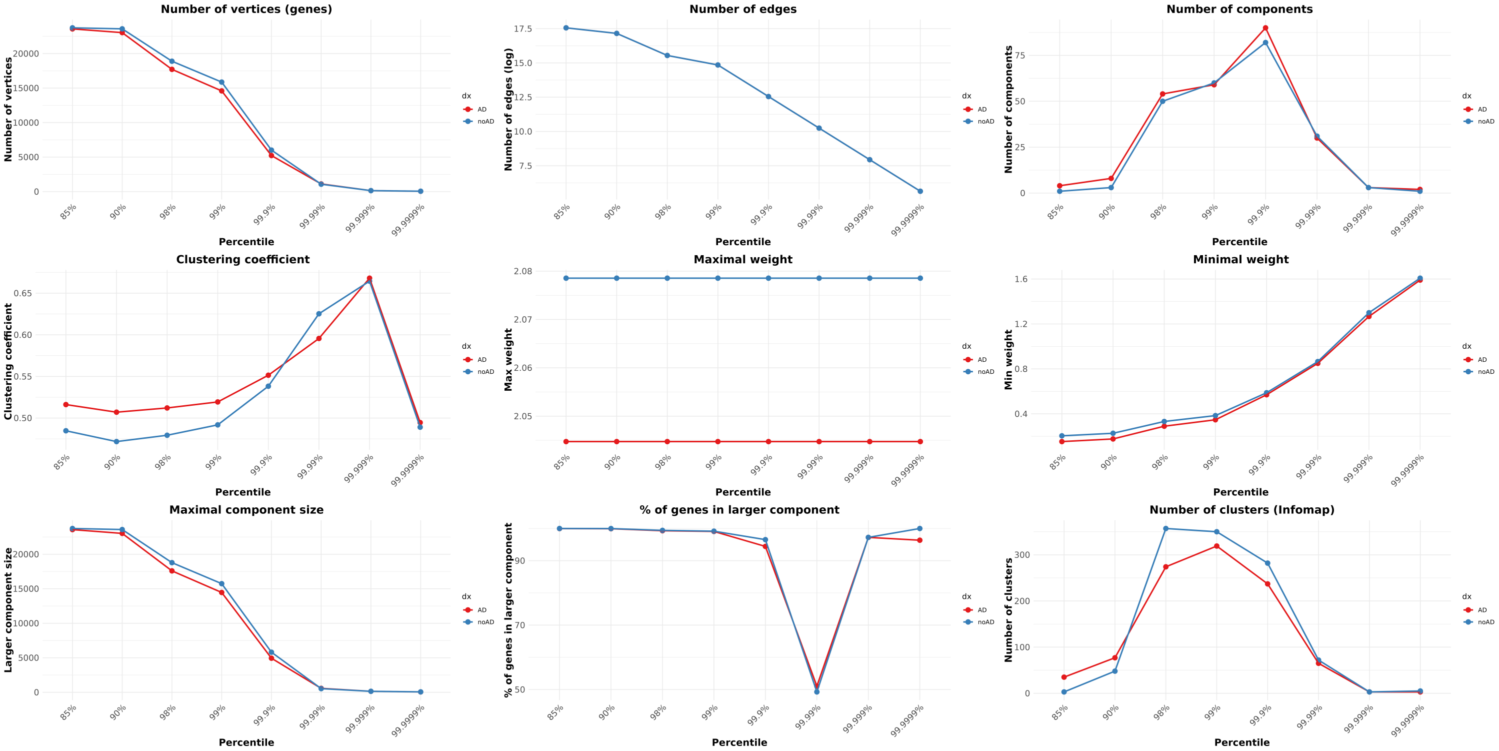

Figure S 2: Various network metrics across different percentiles, comparing two conditions. Subsets of genes are progressively filtered based on Mutual Information thresholds, naturally reducing the number of vertices (genes). The number of edges follows a decline, with a sharper drop at higher percentiles.

3 Goodness-of-fit test

To assess the degrees distribution type in both networks, a fit analysis to multiple statistical distributions was performed. Data from networks constructed from mutual information measures were used, considering a filtering threshold at the 99.99th percentile.

To determine which distribution best describes the degree distribution in both networks, six theoretical distributions were fitted. `"MASS"` [28], `"fitdistrplus"` [8], `"VGAM"` [32], and `"poweRlaw"` [14] R packages were used to fit distributions .

- **Power-Law**: The threshold parameter $X_{\min}$ was estimated using the maximum likelihood method and the fit was validated using a resampling procedure with 500 simulations.
- **Poisson distribution**: The distribution was fitted using the maximum likelihood method to estimate the parameter λ, followed by a Kolmogorov-Smirnov (KS) test to evaluate the fit.
- **Normal (Gaussian) distribution**: The mean and standard deviation of the data were estimated, and the fit was validated with the KS test.
- **Exponential distribution**: The decay rate parameter λ was estimated, and its fit was evaluated using the KS test.

Tables S1 and S2 show p-values of the distribution fitting.

Distribution	p-value
Power-Law	1.8×10^{-1}
Poisson	1.136757×10^{-39}
Gaussian	1.145851×10^{-1}
Exponential	$4.033584 \times 10^{-194}$

Table S 1: P-values obtained from the Kolmogorov-Smirnov test for different fitted degree distributions in the control network. A higher p-value suggests a better fit to the observed data.

Distribution	p-value
Power-Law	2.6×10^{-1}
Poisson	1.72×10^{-44}
Gaussian	2.84×10^{-2}
Exponential	5.46×10^{-195}

Table S 2: P-values obtained from the KS test for different fitted degree distributions in the AD network. A higher p-value suggests a better fit to the observed data.

As the higher p value was the fitted by the Power-Law distribution (1.8×10^{-1} and 2.6×10^{-1} for the control and AD network, respectively), the degree distribution of both networks looks more like a Power-Law distribution, suggesting that the network has a scale-free structure with the presence of hubs.

4 Null model

To evaluate the non-random structure of gene expression networks associated with Alzheimer's disease (AD) and the control group, a null model was applied by randomizing the networks while maintaining the original degree distribution. We generated 1,000 randomized versions of each network, and for each randomized network we calculated three metrics: clustering coefficient, assortativity,

and modularity. For each metric, a bootstrapping approach was applied on the distribution of the randomized networks to calculate confidence intervals and determine the significance of the observed metrics (See FigureS3). For the Clustering coefficient, the assortativity and the Modularity the degree distribution was maintained. For the Scaling coefficient gamma γ, by randomizing edges without maintaining the degree distribution. 95% confidence intervals were calculated for the clustering coefficient, assortativity and modularity in the AD and control networks. In both cases, the metrics observed in the real networks were outside the confidence intervals, indicating behavior different from that expected by chance (see TableS3). Both networks exhibit non-random clustering, assortativity and modularity reflecting a densely connected modular or community structure typical of biological networks, where related genes tend to cluster together.

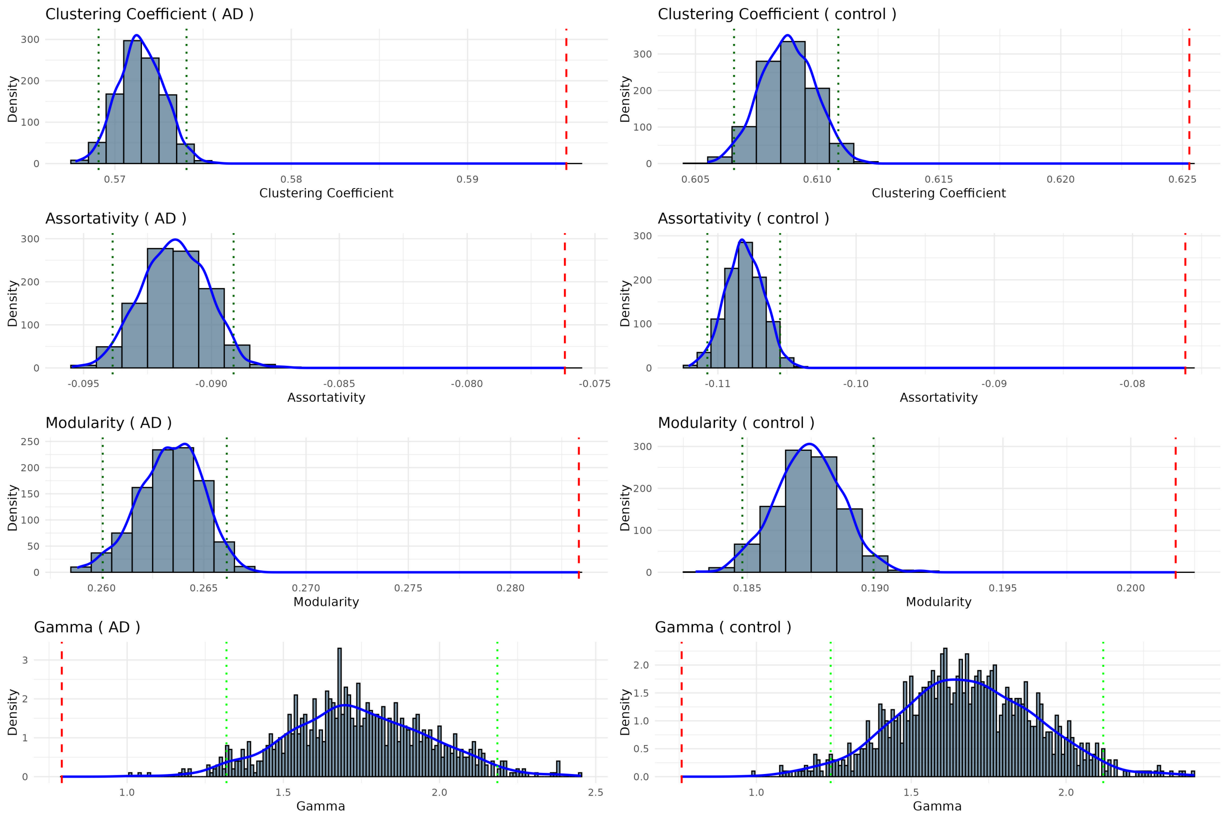

Figure S 3: For the Clustering coefficient, the assortativity and the Modularity the degree distribution was maintained. For the Scaling coefficient gamma γ, edges were randomized without maintaining the degree distribution. Red dashed lines indicate the empirical value for each characteristic. Green dashed lines indicate the random network distributions confidence intervals. All empirical values fall well outside the range of the confidence intervals. **(Top)** Clustering Coefficient. **(Middle)** Assortativity. **(Bottom)** Modularity.

5 Hub genes and high betweenness centrality genes

Both graphs have different genes central for connectivity. Some genes are functioning exclusively as high centrality genes in a network in specific. TableS3 shows the description of the hub genes that are exclusively hubs in the AD network. FigureS4 shows differences in betweenness centrality of specific nodes to the graphs.

Table S 3: Description of the hub genes that are exclusively hubs in the AD network

**Gene**	**Chromosome**	**Degree in AD network**	**Degree in control network**	**Description**
KRT6B	12	207	165	Keratin 6B is a type II cytokeratin, one of a number of isoforms of keratin 6. KRT6B has been highly associated with bladder cancer [22]. Other genes of the KRT family have been associated with AD [30].

Gene	Chromosome	Degree in AD network	Degree in control network	Description
COL6A5	3	199	163	COL6A5 encodes the α4(VI) chains of Collagen VI (Fitzgerald et al., 2013). Type VI collagen contributes to the structural stability of the neuronal environment [29] Increased expression of collagen VI in neurons has been reported to protect against Aβ toxicity. It is known that excitatory neurons are likely to acquire a collagenolytic phenotype during cellular senescence, characterized by down-regulated expression of collagen components, including COL6A5 [16]. It would be interesting to identify the genes that are co-expressed with COL6A5 and analyze their functions to seek for mechanisms related to resilience.

Gene	Chromosome	Degree in AD network	Degree in control network	Description
PDCD5P1	12	189	154	PDCD5P1 is the pseudogene 1 of PDCD5. It has been reported that PDCD5 may play a role in programmed cell death observed in neurodegenerative disorders [5]. In a plasma protein association study with AD, a concordance was observed between proteins known to be mutated or deregulated in AD patient brains and some proteins observed in AD patient plasma from endogenous peptides, including PDCD5 [11]
H3C9P	6	184	152	H3C9P is a pseudogene of H3C9, an H3 Clustered Histone. There is no further information on the relationship of this gene to Alzheimer's disease. [17]

Gene	Chromosome	Degree in AD network	Degree in control network	Description
FCRL1	1	179	166	FCRL1 encodes other member of the immunoglobulin receptor superfamily [9]. FCRL1 and FCRL3 are selectively expressed in different lineages of human B cells, while the expression of FCRL3 has also been detected in the natural killer (NK) cells and human T-cells [10]. FCRL1 has been identified as a down-regulated gene in DEA when comparing AD vs normal controls [7]. Also, an analysis on GWAS data for late-onset AD based in Gaussian causal mixture modelling (MiXeR) identified FCRL1 as a genomic loci jointly associated with Alzheimer's disease (AD) and multiple sclerosis (MS) [12].

Gene	Chromosome	Degree in AD network	Degree in control network	Description
ONECUT3	19	176	164	Transcription factor ONECUT3 regulates chromatin accessibility in neurons [26]. Also, it plays a role in cell differentiation and metabolism [23]. Other paralog genes of the ONECUT family were found to regulate long non-coding RNAs involved in the pathogenesis of Alzheimer's disease [24]
H2BC14	6	174	137	H2BC14 encodes a replication-dependent histone that is a member of the histone H2B family. It;s a core component of nucleosome. Transcriptomic analysis revealed alterations in the expression of H2BC14 after treatment with a complex composed of Moringina (MOR) encapsulated in α-cyclodextrin (α-CD), compared to cells exposed to the amyloid-β [21]

Gene	Chromosome	Degree in AD network	Degree in control network	Description
CCL18	17	174	139	CCL18, is part of a group of beta-chemokines linked to TH2-driven immune responses [4]. It carries out a range of functions, including mild chemotactic activity, immune regulation, promotion of fibrosis, and facilitation of cancer progression. Previously, CCL18 was proposed as part of a plasma biomarker panel for identifying Alzheimer's disease, although no significant relationship has been established [3].
H2AC4	6	172	138	This gene encodes a replication-dependent histone member of the histone H2A family [17].
CROCC2	2	170	104	CROCC2 encodes a 1480-amino acid protein. This gene has a 55% of similarity to CROCC [13] a structural component of the ciliary rootlet which contributes to centrosome [2]. It has been identified as a down-regulated gene in DEA when comparing AD vs normal controls [27].

Gene	Chromosome	Degree in AD network	Degree in control network	Description
SLC25A24P1	1	164	151	SLC25A24P1 is the pseudogene 1 of SLC25A24, which encodes a calcium-binding mitochondrial carrier protein from the solute carrier family 25. SLC25A24 enables the electro-neutral and reversible exchange of ATP-Mg/Pi and phosphate between the cytosol and mitochondria, which is necessary for maintaining optimal adenine nucleotide levels in the mitochondrial matrix. Mutations in SLC25A24 disrupt mitochondrial ATP synthesis, resulting in hyperpolarization, increased proton leakage, and impaired energy metabolism. These mutations are associated with Fontaine syndrome, a condition characterized by features of premature aging [31]. No further associations with AD have been made with this gene.

**Gene**	**Chromosome**	**Degree in AD network**	**Degree in control network**	**Description**
PDE4DIP	1	161	158	PDE4DIP is the psuedogene of PDE4DI, a centrosome/Golgi-associated protein, which interacts with cyclic nucleotide phosphodiesterases. It has been identified as a switch gene in multiple dementia types [18]. Switch genes control the timing and duration of expression in other genes [1]. In particular, specific gene-switching events have been observed in the progression from healthy aging to Alzheimer's disease in brain regions such as the hippocampus and posterior cingulate cortex ([18]

**Gene**	**Chromosome**	**Degree in AD network**	**Degree in control network**	**Description**
FCRL3	1	159	125	FCRL3 encodes a glycoprotein that is a member of the immunoglobulin receptor superfamily [19]. This gene promotes TLR9-induced B cell activation and suppresses plasma cell differentiation [15] and it's related to a variety of immune disorders [6]. Suggestive differences between APOE2 and APOE3 carriers have been observed in cognitive impaired subjects for FCRL3 [20]. Additionally, FCRL3 has been identified as an Alzheimer's disease-related protein through an iterative optimization algorithm that analyzed symptoms and protein similarities across AD-related diseases [33]

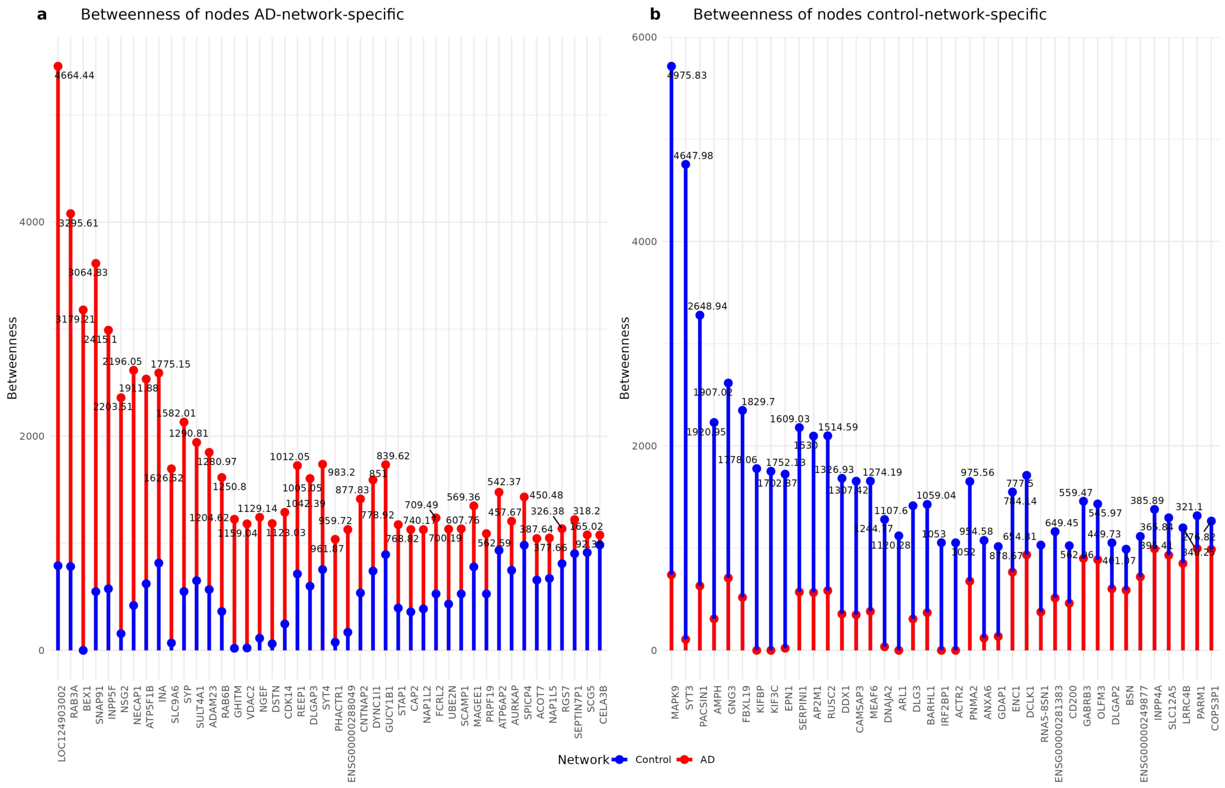

Figure S 4: Changes in the betweenness of the high betweennes centrality nodes specific to the AD network (a) and to the control network (b). Numbers above lines indicate the differences in betweenness to both graphs.

5.1 Modularity per module

To explore whether the global modularity differences arise from local connection reorganization, we calculated Q (See Equation 1) per module for both networks. Specifically, we evaluated the relationship between Q and the number of hubs within each module by constructing subgraphs for each connected group of nodes and calculating their respective modularity values. This approach allowed us to examine whether local network organization contributes to the observed global differences in modularity.

Larger modules exhibit higher modularity because of their greater number of connections, allowing for better distribution. In both of our models, hub genes—defined as the top 10% of genes with the highest co-expression—are found exclusively in the largest module (Module 1), which is also the most modular. While modularity tends to decline as module size decreases, this relationship is not monotonic.

The general modularity differences between the two networks arise primarily from differences in the largest modules. The modularity of the largest module in the control network was 0.17, compared to 0.23 in the AD network, indicating greater compartmentalization. Larger modules not only have higher modularity but also contain more hubs, as a higher number of nodes increases the likelihood of some becoming hubs while also providing more non-hub nodes that can connect to them. These modularity differences align with the observation that the AD network has more hubs, as seen in the degree distribution (**??**).

$$Q = \frac{1}{2m} \sum_{i,j} \left(A_{ij} - \gamma \frac{k_i k_j}{2m} \right) \delta(c_i, c_j) \tag{1}$$

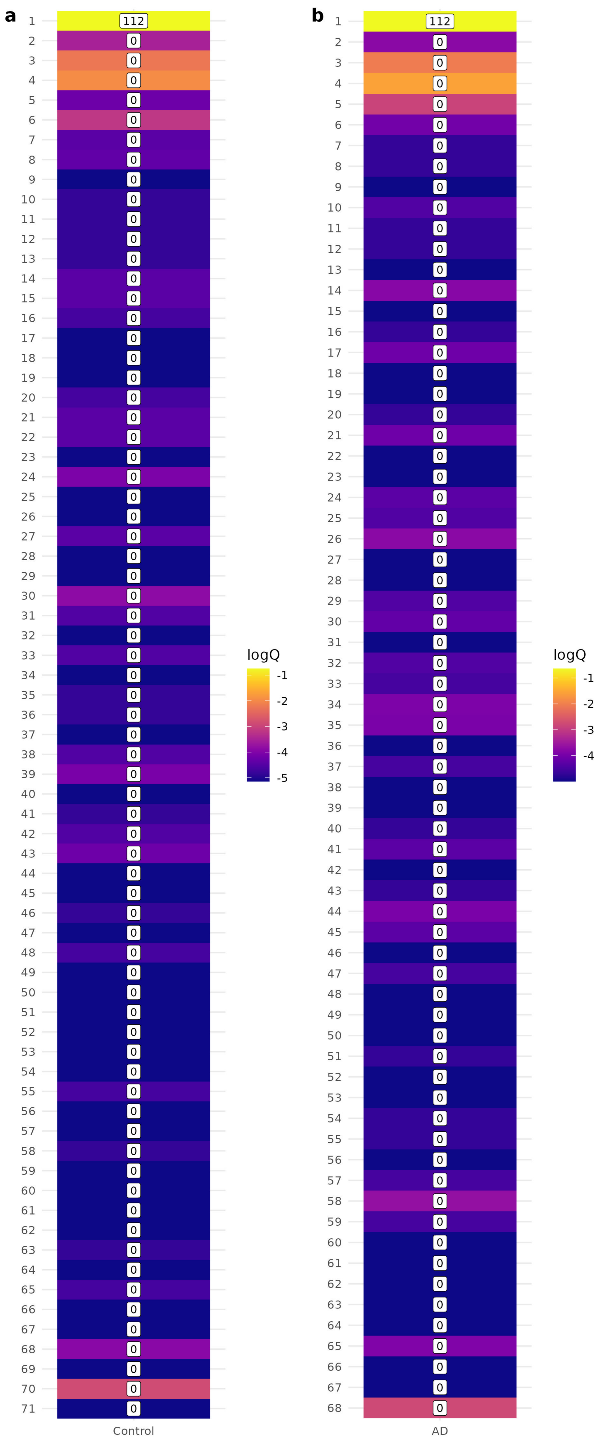

Figure S 5: Distribution of log-transformed Q-values (logQ). in **(a)** Control and **(b)** AD networks. Numbers in the boxes indicate the number of hub genes in the correspondent module.

References

[1] Alberts, B., A. Johnson, J. Lewis, M. Raff, K. Roberts, and P. Walter (2002). How Genetic Switches Work. In *Molecular Biology of the Cell. 4th edition*. Garland Science.

[2] Bahe, S., Y.-D. Stierhof, C. J. Wilkinson, F. Leiss, and E. A. Nigg (2005). Rootletin forms centriole-associated filaments and functions in centrosome cohesion. *The Journal of Cell Biology 171*(1), 27–33.

[3] Björkqvist, M., M. Ohlsson, L. Minthon, and O. Hansson (2012). Evaluation of a Previously Suggested Plasma Biomarker Panel to Identify Alzheimer's Disease. *PLoS ONE 7*(1), e29868.

[4] Bonecchi, R., G. Bianchi, P. P. Bordignon, D. D'Ambrosio, R. Lang, A. Borsatti, S. Sozzani, P. Allavena, P. A. Gray, A. Mantovani, and F. Sinigaglia (1998). Differential Expression of Chemokine Receptors and Chemotactic Responsiveness of Type 1 T Helper Cells (Th1s) and Th2s. *Journal of Experimental Medicine 187*(1), 129–134.

[5] Burté, F., V. Carelli, P. F. Chinnery, and P. Yu-Wai-Man (2015). Disturbed mitochondrial dynamics and neurodegenerative disorders. *Nature Reviews. Neurology 11*(1), 11–24.

[6] Chistiakov, D. A. and A. P. Chistiakov (2007). Is FCRL3 a new general autoimmunity gene? *Human Immunology 68*(5), 375–383.

[7] Cohen, D., A. Pilozzi, and X. Huang (2020). Network Medicine Approach for Analysis of Alzheimer's Disease Gene Expression Data. *International Journal of Molecular Sciences 21*(1), 332.

[8] Delignette-Muller, M. L. and C. Dutang (2015). fitdistrplus: An R package for fitting distributions. *Journal of Statistical Software 64*(4), 1–34.

[9] DeLuca, J. M., M. K. Murphy, X. Wang, and T. J. Wilson (2021). FCRL1 regulates BCR-induced ERK activation through GRB2. *Journal of immunology (Baltimore, Md. : 1950) 207*(11), 2688.

[10] Ehrhardt, G. R. A. and M. D. Cooper (2011). Immunoregulatory roles for fc receptor-like molecules. *Current Topics in Microbiology and Immunology 350*, 89–104.

[11] Florentinus-Mefailoski, A., P. Bowden, P. Scheltens, J. Killestein, C. Teunissen, and J. G. Marshall (2021). The plasma peptides of Alzheimer's disease. *Clinical Proteomics 18*, 17.

[12] Fominykh, V., A. A. Shadrin, P. P. Jaholkowski, S. Bahrami, L. Athanasiu, D. P. Wightman, E. Uffelmann, D. Posthuma, G. Selbæk, A. M. Dale, S. Djurovic, O. Frei, and O. A. Andreassen (2023). Shared genetic loci between Alzheimer's disease and multiple sclerosis: Crossroads between neurodegeneration and immune system. *Neurobiology of Disease 183*, 106174.

[13] Gascoigne, D. K., S. W. Cheetham, P. B. Cattenoz, M. B. Clark, P. P. Amaral, R. J. Taft, D. Wilhelm, M. E. Dinger, and J. S. Mattick (2012). Pinstripe: a suite of programs for integrating transcriptomic and proteomic datasets identifies novel proteins and improves differentiation of protein-coding and non-coding genes. *Bioinformatics 28*(23), 3042–3050.

[14] Gillespie, C. S. (2015). Fitting heavy tailed distributions: The poweRlaw package. *Journal of Statistical Software 64*(2), 1–16.

[15] Li, F. J., D. M. Schreeder, R. Li, J. Wu, and R. S. Davis (2013). FCRL3 promotes TLR9-induced B cell activation and suppresses plasma cell differentiation. *European journal of immunology 43*(11), 10.1002/eji.201243068.

[16] Mavrogonatou, E., H. Pratsinis, A. Papadopoulou, N. K. Karamanos, and D. Kletsas (2019). Extracellular matrix alterations in senescent cells and their significance in tissue homeostasis. *Matrix Biology: Journal of the International Society for Matrix Biology 75-76*, 27–42.

[17] O'Leary, N. A., M. W. Wright, J. R. Brister, S. Ciufo, D. Haddad, R. McVeigh, B. Rajput, B. Robbertse, B. Smith-White, D. Ako-Adjei, A. Astashyn, A. Badretdin, Y. Bao, O. Blinkova, V. Brover, V. Chetvernin, J. Choi, E. Cox, O. Ermolaeva, C. M. Farrell, T. Goldfarb, T. Gupta, D. Haft, E. Hatcher, W. Hlavina, V. S. Joardar, V. K. Kodali, W. Li, D. Maglott, P. Masterson,

K. M. McGarvey, M. R. Murphy, K. O'Neill, S. Pujar, S. H. Rangwala, D. Rausch, L. D. Riddick, C. Schoch, A. Shkeda, S. S. Storz, H. Sun, F. Thibaud-Nissen, I. Tolstoy, R. E. Tully, A. R. Vatsan, C. Wallin, D. Webb, W. Wu, M. J. Landrum, A. Kimchi, T. Tatusova, M. DiCuccio, P. Kitts, T. D. Murphy, and K. D. Pruitt (2016). Reference sequence (RefSeq) database at NCBI: current status, taxonomic expansion, and functional annotation. *Nucleic Acids Research 44*(D1), D733–745.

[18] Potashkin, J. A., V. Bottero, J. A. Santiago, and J. P. Quinn (2020). Bioinformatic Analysis Reveals Phosphodiesterase 4D-Interacting Protein as a Key Frontal Cortex Dementia Switch Gene. *International Journal of Molecular Sciences 21*(11), 3787.

[19] Ravetch, J. V. and S. Bolland (2001). IgG Fc receptors. *Annual Review of Immunology 19*, 275–290.

[20] Shue, F., L. J. White, R. Hendrix, J. Ulrich, R. L. Henson, W. Knight, Y. A. Martens, N. Wang, B. Roy, S. C. Starling, Y. Ren, C. Xiong, Y. W. Asmann, J. A. Syrjanen, M. Vassilaki, M. M. Mielke, J. Timsina, Y. J. Sung, C. Cruchaga, D. M. Holtzman, G. Bu, R. C. Petersen, M. G. Heckman, and T. Kanekiyo (2024). CSF biomarkers of immune activation and Alzheimer's disease for predicting cognitive impairment risk in the elderly. *Science Advances 10*(14), eadk3674. Publisher: American Association for the Advancement of Science.

[21] Silvestro, S., L. Chiricosta, A. Gugliandolo, R. Iori, P. Rollin, D. Perenzoni, F. Mattivi, P. Bramanti, and E. Mazzon (2021). The Moringin/-CD Pretreatment Induces Neuroprotection in an In Vitro Model of Alzheimer's Disease: A Transcriptomic Study. *Current Issues in Molecular Biology 43*(1), 197–214.

[22] Song, Q., H. Yu, Y. Cheng, J. Han, K. Li, J. Zhuang, Q. Lv, X. Yang, and H. Yang (2022). Bladder cancer-derived exosomal KRT6B promotes invasion and metastasis by inducing EMT and regulating the immune microenvironment. *Journal of Translational Medicine 20*, 308.

[23] Sunita Prajapati, K., S. Gupta, S. Chaudhri, and S. Kumar (2024, May). Role of ONECUT

family transcription factors in cancer and other diseases. *Experimental Cell Research 438*(1), 114035.

[24] Tang, L., L. Liu, G. Li, P. Jiang, Y. Wang, and J. Li (2019). Expression Profiles of Long Non-coding RNAs in Intranasal LPS-Mediated Alzheimer's Disease Model in Mice. *BioMed Research International 2019*, 9642589.

[25] Tarazona, S., P. Furió-Tarí, D. Turrà, A. D. Pietro, M. J. Nueda, A. Ferrer, and A. Conesa (2015). Data quality aware analysis of differential expression in RNA-seq with NOISeq R/Bioc package. *Nucleic Acids Research 43*(21), e140.

[26] van der Raadt, J., S. H. C. van Gestel, N. Nadif Kasri, and C. A. Albers (2019). ONECUT transcription factors induce neuronal characteristics and remodel chromatin accessibility. *Nucleic Acids Research 47*(11), 5587–5602.

[27] Vastrad, B. and C. Vastrad (2021). Identification of Key Pathways and Genes in Dementia via Integrated Bioinformatics Analysis.

[28] Venables, W. N. and B. D. Ripley (2002). *Modern Applied Statistics with S* (Fourth ed.). New York: Springer. ISBN 0-387-95457-0.

[29] Wareham, L. K., R. O. Baratta, B. J. D. Buono, E. Schlumpf, and D. J. Calkins (2024). Collagen in the central nervous system: contributions to neurodegeneration and promise as a therapeutic target. *Molecular Neurodegeneration 19*, 11.

[30] Wong, Y. Y., L. Y. Xiong, D. K. Mori-Fegan, S. Noor, M. J. Chenoweth, S. S. Mirza, M. Masellis, S. E. Black, W. Swardfager, and Alzheimer's Disease Neuroimaging Initiative (2023). Relationships between polymorphisms in keratin genes and Alzheimer's disease phenotypes. *Alzheimer's & Dementia 19*(S12), e074162.

[31] Writzl, K., A. Maver, L. Kovačič, P. Martinez-Valero, L. Contreras, J. Satrustegui, M. Castori, L. Faivre, P. Lapunzina, A. B. Van Kuilenburg, S. Radović, C. Thauvin-Robinet, B. Peterlin,

A. Del Arco, and R. C. Hennekam (2017). De Novo Mutations in SLC25A24 Cause a Disorder Characterized by Early Aging, Bone Dysplasia, Characteristic Face, and Early Demise. *The American Journal of Human Genetics 101*(5), 844–855.

[32] Yee, T. W. and C. J. Wild (1996). Vector generalized additive models. *Journal of Royal Statistical Society, Series B 58*(3), 481–493.

[33] Zhao, T., Y. Hu, T. Zang, and Y. Wang (2020). Identifying Protein Biomarkers in Blood for Alzheimer's Disease. *Frontiers in Cell and Developmental Biology 8*, 472.
